# Primary adrenal melanoma with extensive metastasis: a case report and literature review

**DOI:** 10.3389/fonc.2025.1654218

**Published:** 2025-08-14

**Authors:** Qi Guo, Dai Shi, Guilai Li, Yue Liu, Guoping Xu

**Affiliations:** ^1^ Department of Radiology, Second Hospital of Tianjin Medical University, Tianjin, China; ^2^ Department of Radiology, Second Affiliated Hospital of Soochow University, Suzhou, China

**Keywords:** adrenal gland, melanoma, diagnosis, metastasis, primary

## Abstract

Primary adrenal melanoma is an exceedingly rare diagnosis. We present a case of primary left adrenal melanoma with rapid and extensive distant metastasis. The patient underwent a biopsy of the left adrenal tumor, and pathological analysis confirmed malignant melanoma. Immunohistochemical staining revealed positive cytoplasmic reactivity for Melan A, HMB45, and SOX-10. We also review relevant cases reported in the English literature and discuss the diagnostic criteria, differential diagnoses, and potential management strategies.

## 1 Introduction

The adrenal gland is the prevalent site for metastatic disease. Malignant melanoma being one of the most common cancers that metastasize to the adrenal glands (1, 2). However, primary adrenal melanoma (PAM) is an exceedingly rare entity, with only a limited number of cases reported in the literature (3). The clinical manifestations of PAM are nonspecific, usually presenting with symptoms such as pain and weight loss—features common to many other tumors (4). The main diagnostic challenge lies in determining whether it is a primary adrenal tumor. Additionally, it is necessary to distinguish PAM from other primary adrenal tumors, especially pigmented pheochromocytomas. Therefore, enhancing the overall understanding of PAM is essential, especially in terms of diagnosis, to help reduce clinical misdiagnosis and subsequent inappropriate treatment strategies.

## 2 Case presentation

A 37-year-old previously healthy Chinese man presented to our hospital with a 1-month history of left-sided lower back pain of unknown etiology. His family history was unremarkable, and physical examination revealed no abnormalities. He denied endocrine abnormalities, weight loss, nausea or vomiting, chills or high fever, discomfort such as urinary frequency, urgency or pain.

**Figure 1:**
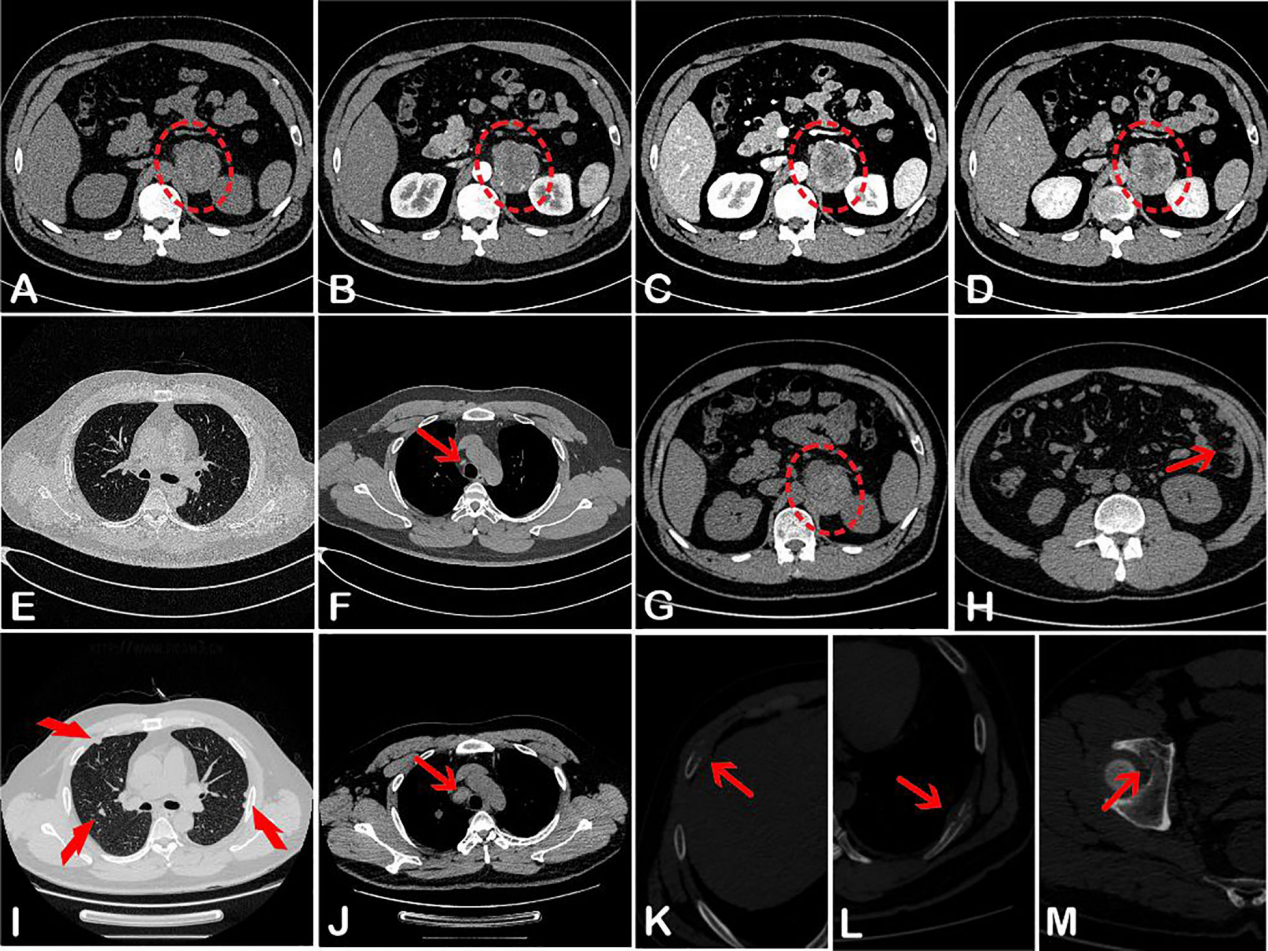
Abdominal CT **(A–D)** on 7 March 2024, showed a left-sided heterogeneous adrenal lesion (red oval) with uneven enhancement. Corresponding CT attenuation values were 35–49 HU, 37–73 HU, 35–86 HU, and 36–89 HU, respectively. Chest CT **(E, F)** revealed only a few small lymph nodes adjacent to the aorta (red arrow). One month later, pelvic-abdominal CT **(G, H)** showed a slightly enlarged lesion in the left adrenal region (red oval), along with multiple nodules in the omentum and peritoneum (red arrow) and small lymph nodes surrounding the abdominal aorta. The second chest CT displayed multiple pulmonary nodules **(I)**, markedly enlarged para-aortic lymph nodes **(J)**, and bony destruction of the ribs on both sides **(K, L)**. Additional bone destruction was noted in the right acetabulum **(M)**.

A computed tomography (CT) scan of the adrenal glands performed on 7 March 2024 (**Figures 1A–D**), showed a heterogeneous mass in the region of the left adrenal gland, measuring approximately 5.4 cm × 4.4 cm at its largest cross-section. The mass showed inhomogeneous enhancement with areas of necrosis. No mesenteric lymph node enlargement or peritoneal lesions were observed. Additionally, there were no signs of malignancy in the thoracic region (**Figures 1E, F**).

Laboratory investigations following admission showed normal levels of serum sodium, potassium, cortisol, adrenocorticotropic hormone, aldosterone, angiotensin II, serum catecholamines, epinephrine, norepinephrine, and 24-h urinary free cortisol. Both blood and urine routine tests were unremarkable. There was no clinical evidence of hyper- or hypoadrenocorticism. Notably, the patient endorsed that the low back pain resolved spontaneously during this period.

One month later, the patient sought medical attention again, claiming that the lower back pain had worsened. A follow-up pelvic-abdominal CT scan showed that the tumor in the left adrenal region had slightly enlarged, measuring approximately 5.9 cm × 4.7 cm (**Figure 1G**). Additionally, multiple nodules had appeared in the omentum and peritoneum, along with small lymph nodes around the abdominal aorta and the presence of abdominal effusion (**Figure 1H**). A repeat chest CT (**Figures 1I–L**) revealed multiple pulmonary nodules, significantly enlarged para-aortic lymph nodes, and bilateral rib bone destruction. Bone destruction was also noted in the right acetabulum (**Figure 1M**). Whole-body bone scintigraphy confirmed the rib and acetabular lesions observed on CT.

**Figure 2:**
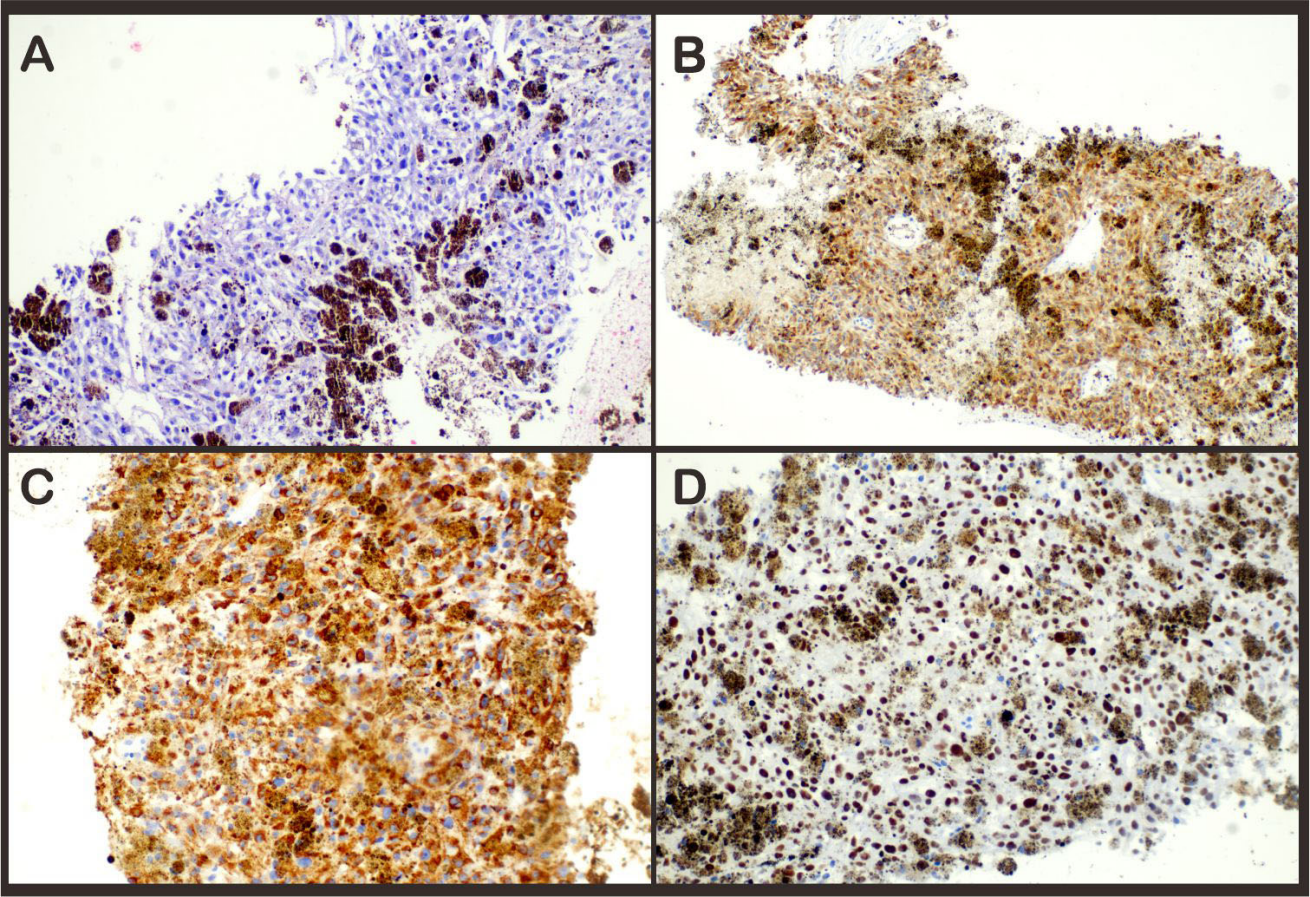
Hematoxylin–eosin stain **(A)** revealed infiltration of melanoma into the adrenal gland. Immunohistochemical staining demonstrated expression of **(B)** Melan-A, **(C)** HMB45, and **(D)** SOX-10 expression in the tumor; magnification ×20.

Given the extent of metastatic disease, the patient was deemed unsuitable for surgical resection. With the consent of the patient, a left adrenal tumor was performed on 10 April. Histological analysis confirmed a diagnosis of malignant melanoma of the adrenal gland. Immunohistochemical staining showed strong cytoplasmic positivity for Melan A, HMB-45, and SOX-10 (**Figure 2**).

Following histopathological confirmation, a comprehensive re-evaluation of the patient’s medical history and physical examination was conducted, with specific focus on potential skin, tongue, mucosal, and ocular lesions. No suspicious melanotic lesions were identified, and there was no history of prior skin lesion excision. Based on the clinical presentation and histopathological findings, the diagnosis of primary adrenal malignant melanoma with widespread metastases was established.

**Figure 3:**
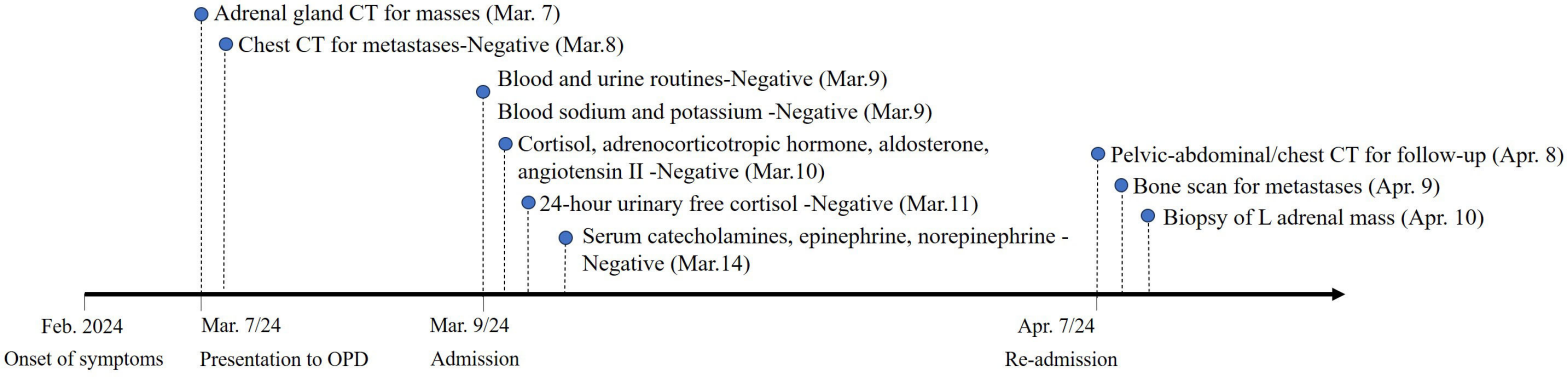
Timeline with data.

Please refer to the schedule (**Figure 3**) for a timeline of relevant examinations and management data.

## 3 Discussion

Malignant melanoma is an aggressive neoplasm resulting from uncontrolled growth of melanocytes. It is prone to distant metastasis, including to the adrenal glands, and generally has a poor prognosis (5, **6**). It most commonly originates from the skin, but can also be found in the oral mucosa, larynx, ciliary body, choroid plexus, iris, respiratory tract, digestive tract, and other mucosal or visceral sites (7). Melanoma involving the adrenal gland is typically metastatic, originating from a primary tumor elsewhere. In contrast, PAM is defined as a melanocytic tumor confined to the adrenal gland without evidence of an extra-adrenal primary lesion, which is exceptionally rare and usually presents as an incidental nonfunctional mass (8). Non-specific symptoms like pain, are the most common clinical manifestation of PAM, as seen in our case. The patient we reported initially presented with low back pain, which often leads to delayed diagnosis due to overlap with other adrenal disorders.

The diagnosis of malignant melanoma is mainly based on invasive pathological and immunohistochemical examination. While electron microscopy can identify characteristic melanosomes, immunohistochemistry is especially crucial for cases lacking visible pigmentation or containing only sparse melanin granules. Markers such as Melan-A, HMB-45, SOX-10, and MITF have high specificity for melanocytic tumors (9, 10). In our case, strong positivity for these markers—alongside the absence of neuroendocrine marker expression—supported the diagnosis of PAM.

The pathogenesis of PAM remains unclear yet. However, current theories suggest that it may originate from neural crest-derived cells within the adrenal medulla (11). These pluripotent neural crest cells possess migratory capability and can differentiate into melanocytes or chromaffin cells. It is hypothesized that ectopic melanocytes may appear within the adrenal medulla and, upon malignant transformation, give rise to PAM.

Noninvasive imaging modalities such as CT and MRI often fail to distinguish PAM from other adrenal tumors due to nonspecific features (12, 13). On CT, PAM typically presents as a unilateral, heterogeneous mass with irregular enhancement and infrequent calcification—features indistinguishable from adrenal carcinoma or metastatic disease, as observed in our case. MRI offers limited diagnostic advantage unless the tumor is melanin-rich: small, pigmented tumors may exhibit T1 hyperintensity and T2 hypointensity due to the paramagnetic effects. Whereas large or less pigmented melanomas with necrosis or hemorrhage often display mixed, non-specific signals.

The criteria established by Carsten et al. in 1984 remain the authoritative standard for distinguishing primary adrenal melanoma from metastatic melanoma following a pathological diagnosis of melanoma. These criteria are essential for guiding subsequent clinical management. The key requirements include: (1) solitary involvement of the adrenal gland; (2) no history of prior melanoma or pigmented lesions in typical sites (skin, mucosa, or eyes); (3) no previous excision of pigmented skin or ocular lesions; and (4) ideally, autopsy to rule out occult primaries (14). In our case, the patient clearly met the first three criteria. A comprehensive clinical evaluation—including skin, mucosal, and ocular examination, along with whole-body imaging—revealed no evidence of an extra-adrenal primary lesion. Although the rapid progression of metastases is not pathognomonic, it is consistent with the aggressive biology of PAM. While an autopsy was not performed, current medical practice accepts thorough clinical, imaging (e.g., PET-CT), and endoscopic evaluation as sufficient to exclude hidden primaries in most cases.

An important differential diagnosis for PAM is pigmented pheochromocytoma, which may contain melanin-like pigment or actual melanin (15). Immunohistochemical staining and electron microscopy can help differentiate the two: pigmented pheochromocytomas typically express neuroendocrine markers (e.g., synaptophysin and chromogranin) and contain neurosecretory granules (16), which are absent in malignant melanoma. Although HMB-45 may occasionally be positive in some medullary neoplasms, malignant melanomas consistently lack neuroendocrine marker expression.

In our case, several findings supported the diagnosis of PAM: (1) a negative workup for extra-adrenal primary tumors despite exhaustive clinical and radiological evaluation; (2) histopathological features and strong melanocytic marker positivity; and (3) absence of clinical or laboratory evidence of endocrine dysfunction, such as catecholamine excess, which is characteristic of pheochromocytoma. Furthermore, the rapid progression to widespread metastases within 1 month of initial presentation aligns more closely with advanced melanoma than with pheochromocytoma.

Currently, no standard treatment protocol is available due to the rarity of PAM. Surgical resection followed by postoperative adjuvant therapy is the treatment strategy for primary or oligometastatic adrenal melanoma. For patients undergoing surgical treatment, the complete tumor, the contralateral adrenal gland, and the affected kidney are usually removed. Adjuvant therapies options for malignant melanoma include chemotherapy, immunotherapy, targeted therapy, and radiation therapy (17). Among these, the development of immunotherapies (e.g. interferon (IFN) alpha-2b, interleukin (IL)-2, and PD-1 inhibitors) and targeted therapies (e.g. BRAF/MEK-targeted therapy) has greatly improved progression-free survival and melanoma-specific survival of patients with advanced melanoma (18, 19).

## 4 Conclusion

In conclusion, PAM is extremely rare but highly malignant tumor in clinical practice. Patients with PAM typically present with an adrenal mass and non-specific symptoms such as pain, weight loss. Therefore, a comprehensive examination including skin examination, funduscopy, gastroenteroscopy, radiological examination) necessary to rule out metastases. The diagnosis is currently mainly relies on pathology and immunohistochemistry (e.g., Melan-A/HMB-45 positivity). Standardized treatment regimens have not yet been identified, and treatment protocols are often extrapolated from cutaneous melanoma. 

## Data Availability

The raw data supporting the conclusions of this article will be made available by the authors, without undue reservation.
